# Genetic analyses of brown hare (*Lepus europaeus*) support limited migration and translocation of Greek populations

**DOI:** 10.1371/journal.pone.0206327

**Published:** 2018-10-31

**Authors:** Styliani Minoudi, Ioannis Papapetridis, Nikoleta Karaiskou, Evangelos Chatzinikos, Costas Triantaphyllidis, Theodore J. Abatzopoulos, Alexandros Triantafyllidis

**Affiliations:** 1 Deparment of Genetics, Development and Molecular Biology, School of Biology, Aristotle University of Thessaloniki, Thessaloniki, Greece; 2 4th Hunting Federation of Sterea Hellas, Fokionos 8 and Ermou, Athens, Greece; University of Warsaw, POLAND

## Abstract

Numerous studies have shown that the phylogeography of many species, including European brown hare, has been affected by the climatic oscillations of the Pleistocene. During this period the Balkans acted as a major refugium offering habitable conditions for many species. However, few studies have focused on the specific role of the Greek peninsula in the phylogeographic history of species in this southernmost margin of Balkans. We, therefore analyzed a 528 bp fragment of the D-loop region of mitochondrial DNA in 154 wild brown hare individuals from unsampled areas from both mainland and island Greece and compared it to 310 available brown hare sequences (including 110 Greek samples). Newly identified haplotypes show characteristic distribution in specific Greek areas reinforcing the theory that Greece can be considered as a subrefuge within Balkans for a number of species, with several “refugia within refugia” spots, holding significant genetic diversity. No haplotypes from wild Greek individuals clustered with the Central and Western Europe group revealing a minimal contribution of this area to the colonization of central Europe. One hundred and ten reared brown hares were also analyzed to elucidate the impact of introductions on local populations. Most of these samples presented close genetic affinity with haplotypes from Central and Western Europe indicating that farms in Greece use breeders from those areas. Therefore, despite human translocation of individuals, the genetic structure of brown hare has mostly been influenced by paleoclimatic conditions and minimally by human actions.

## Introduction

Pleistocene glaciation cycles have considerably influenced the current geographic distribution of many European temperate species. During the Last Glacial Maximum (LGM, 26–19 ka, [1]), large parts of Europe presented inhospitable habitats and diverse species were limited to warmer regions of Europe [2, 3]; the southern peninsulas of Italy, Iberia and Balkans (extending into Asia Minor) provided more habitable conditions and acted as major refugia for many species [4–6]. The populations that survived in these isolated regions underwent limited gene exchanges resulting in independent evolution and genetic divergence. According to this theory, these separate refugia contributed significantly to the post-glacial recolonization of the rest of Europe [2, 7, 8]. More recent evidence proves that many species also survived in unexpected latitudes in northern or cryptic refugia [3, 9–11]. Such refugia have been identified in regions of Western Europe, Central Europe and the Carpathians [8, 12–14]. “Refugia within refugia” have been identified even within the three Mediterranean peninsulas for animal as well as plant species [15–18].

Pleistocene climate changes are believed to have, also, profoundly affected the phylogeographic structure and the evolutionary history of European brown hare (*Lepus europaeus*). This mammal presents considerable plasticity evidenced by its wide distribution in a range of environments, while it has also been effectively introduced, even in tropical habitats [19]. Its mobility can differ between the two genders (females present a more philopatric behavior, [20]) and between different periods of the year (higher levels of mobility in breeding seasons, [21]). According to previous studies the current genetic structure of European populations is the result of its postglacial spread from the Balkans and Asia Minor [22–24]. Both Kasapidis et al. [22] and Stamatis et al. [24] concluded that brown hares from Greece, Asia Minor and Central Europe were separated in two distinct clades, which may correspond to the post-glacial refugia of Balkans and Anatolia. Fickel et al. [23] confirmed these separate clades and identified an additional Italian one.

However, data on the evolutionary history of brown hare in Greece is missing, especially on populations from Central Greece and the Greek islands, which, some of them, have been shown to be connected to mainland Greece due to sea oscillations during glaciations cycles [25]. We analyzed a fragment of the mitochondrial control region sequence of brown hares both of mainland and island Greece and reared brown hares as well, and we combined our data with results from previous studies aiming to three main objectives: (a) highlight the genetic diversity of brown hare in Greece, (b) search for signs of unique/genetically differentiated lineages in agreement with the ‘refugia within refugia’ hypothesis by applying a more extensive sampling of these regions (c) investigate the impact of introduced genomes by reared individuals into the local populations as it known that restocking programs have taken place in many European countries in order to mitigate brown hare population reductions, without always taking into consideration the effect on the local genetic pool [26–28],

## Materials and methods

### Ethics statement

No animals were sacrificed only for the purposes of this study. The wild brown hare samples in this study represent material collected opportunistically (after hunting procedure) from animals’ hunter-harvested by members of the Greek Hunting Federation of Sterea Hellas during the hunting seasons, according to the prerequisites of the Greek Legislation (Ministerial Decision 103305/6093/14-11-2007, FEK 1626/Β/13.8.2008, FEK 1611/Β/2009, FEK 1183/6-8-2010, FEK 1763/4-8-2011). The reared samples included in this study were collected by private veterinarians. No domesticated individual was euthanized during the study and efforts were taken to mitigate animal suffering. There is not an ethics committee in the Aristotle University of Thessaloniki. But, all experimental procedures follow the code of conduct of the Research Committee of the Aristotle University of Thessaloniki. The study was approved through the acceptance of the program by the Research Committee of the Aristotle University of Thessaloniki (Program number: 84430).

### Sample collection and DNA extraction

A total of 154 wild brown hares were collected from various regions of both mainland and island Greece with the assistance of the 4th Hunting Federation of Sterea Hellas. Muscle tissues were obtained during the hunting seasons of 2006 to 2009. Particularly, we conducted a more comprehensive sampling in Central Greece and Aegean islands (including areas which had not been previously sampled or had been poorly sampled) (S1 Table). Additionally, 110 reared individuals were analyzed from four Greek breeding stations (two breeding station from Evia, one from Agrinio and one from Macedonia) (a sloughed piece from animals’ ear was used). Whole genomic DNA was extracted using the protocol of [29] which is based on the chemical compound CTAB.

### PCR amplification and DNA sequencing

A fragment of 528 bp of D-loop (control region, CR) of mitochondrial (mt) DNA (including the entire CR-1) was amplified using primers Le.H-Dloop and Le.L-Dloop [22]. The 5’ ends of the two primers correspond to positions 15423 and 15951 of the complete mtDNA, respectively. The total volume of polymerase chain reaction was 25μl in which 100ng of genomic DNA was amplified, using 0.05 units of Qiagen Taq polymerase, 2mM dNTPs, 5 pmol of each primer, 2.5 mM MgCl_2_ and 2.5 μl of 10 X Reaction Buffer. Thermal cycling amplification conditions were as follows: initial denaturation at 94°C for 3 min, followed by 33 cycles of strand denaturation at 94°C for 1 min, annealing at 50°C for 45 s and primer extension at 72°C for 40 s and a final 3min elongation time at 72°C. The PCR products were purified using the Nucleospin Extra kit (Macherey-Nagel, Duren, Germany) and sequenced by Sanger method from Macrogen Inc. (Seoul, Korea) using an ABI 3730XL DNA Analyzer.

### Sequence analyses

A total of 264 sequences (dataset 1) (Genbank Accession nos: MH842011-MH842084) were aligned using ClustalW [30] with final adjustments by eye. A second dataset was created aiming at a pan-European analysis. Thus, data from the present analysis were combined with 310 CR-1 sequences of *L*. *europaeus* retrieved from GenBank: 72 sequences described in Kasapidis et al. [22], 69 sequences described in Stamatis et al. [24], 109 sequences described in Fickel et al. (direct submission), 42 sequences described in Fickel et al. [23] and 18 sequences described in Antoniou et al. [31]. All above sequences were long enough to retain informative sites for proper downstream analyses. The overlapping region with the new dataset was 335bp.

The best-fit nucleotide substitution model for this dataset was determined using jModelTest 2.1.10 (under the Bayesian Information Criterion, BIC) [32, 33]. The GTR + I + G model [34, 35], was used for Bayesian phylogeny analyses, carried out with Beast 1.8.4 [36]. The Bayesian tree was constructed using a strict clock model and a coalescent tree prior. The analysis was run for 10^8^ Markov Chain Monte Carlo (MCMC) generations, sampled every 10^4^ generations. Convergence of chains was visualized using Tracer 1.6 [37] discarding the first 20% of trees as burn-in. ESS values for all parameters were > 248, larger than the threshold value of 200 identified by Tracer v. 1.6. The trees produced by Beast were then summarized in TreeAnnotator 1.8.4 and visualized in FigTree 1.4.3 [38]. A D-loop sequence of *Lepus timidus* (Accession number: EF515861), was used as outgroup.

Additionally, a median-joining network [39] was constructed using the software Network 5.0.0 and the frequencies of the sequences (Fluxus Technology) assuming equal weights for all mutations and setting the genetic distance parameter *e* to zero in order to restrict the choice of feasible links in the final network.

Genetic variability indices of brown hare populations, i.e. the number of distinct haplotypes (h), haplotype (Hd) and nucleotide diversity (π) values, were estimated using DnaSP 5 [40]. To estimate the haplotype richness by additional extensive sampling in Greece, a rarefaction curve was calculated in RarefactWin [41] and plotted in Microsoft Excel (Office 365).

## Results

A total of 74 different haplotypes were identified among the 264 newly analyzed samples, 82.4% of these have never been detected before. Fifty seven haplotypes were found in wild brown hares and 20 in domestic individuals; only three haplotypes were shared between wild and domestic samples. In this dataset 1, the population of Central-South Greece possesses the highest haplotypic diversity (0.938), whereas North Greece possesses the highest nucleotide diversity values (0.038) (Table 1). Most haplotypes from Aegean islands (9 out of 12) were unique, characterizing each studied island. Additionally, North Greece populations revealed high percentage of (population-specific) haplotypes per number of individuals. As regards reared populations, haplotype and nucleotide diversity values were generally lower compared to the wild ones (Table 1) with breeding station 2 exhibiting remarkably low diversity values (Hd = 0.275, π = 0.004).

**Table 1:** Genetic diversity values for wild brown hares from five Greek areas and Central/West European countries and for domestic brown hares from four Greek breeding stations. Dataset 1 includes new samples from this study, whereas dataset 2 includes additional sequences from GenBank (for references see MM section).

Region	Dataset	N	H	Hp	V (%)	W (%)	Hd (SD)	π (SD)
North Greece	1	16	11	9	68.7	56.2	0.900 (0.062)	0.038 (0.003)
	2	92	66	64	71.7	69.6	0.982 (0.007)	0.034 (0.002)
Central–South Greece	1	89	37	31	41.6	34.8	0.938 (0.016)	0.018 (0.002)
	2	117	60	56	51.3	47.9	0.963 (0.010)	0.022 (0.001)
Central Aegean Islands	1	45	11	8	24.4	17.8	0.855 (0.031)	0.012 (0.002)
	2	61	20	17	32.8	27.9	0.914 (0.019)	0.015 (0.001)
Eastern Aegean Islands	1	1	1	1	100.0	100.0	-	-
	2	12	6	6	50.0	50.0	0.864 (0.064)	0.023 (0.003)
Ionian Islands	1	3	1	1	33.3	33.3	-	-
	2	5	1	1	20.0	20.0	-	-
Total of wild Greek samples	1	154	57	54	37.0	35.1	0.965 (0.006)	0.025 (0.002)
	2	287	149	149	51.9	51.9	0.982 (0.003)	0.030 (0.001)
Central/West Europe	2	841	130	130	15.5	15.5	0.893 (0.009)	0.009 (0.001)
Breeding Station 1	1	19	5	2	26.3	10.5	0.801 (0.046)	0.007 (0.002)
Breeding Station 2	1	60	6	4	10.0	6.7	0.275 (0.074)	0.004 (0.002)
Breeding Station 3	1	20	9	4	45.0	20.0	0.868 (0.050)	0.019 (0.001)
Breeding Station 4	1	11	4	4	36.4	36.4	0.745 (0.098)	0.007 (0.001)
Total of reared samples	1	110	20	17	18.2	15.5	0.704 (0.043)	0.013 (0.001)

n, sample size; h, number of different haplotypes; Hp, population/region specific haplotypes; V, the percentage of the total number of haplotypes/number of individuals; W, the percentage of the population–specific haplotypes/number of individuals; Hd, haplotype diversity; π, nucleotide diversity (SD, Standard deviation)

The combination of our haplotypes and the retrieved sequences (384 sequences in total, corresponding to 1128 individuals) resulted in a dataset of 326 unique haplotypes (dataset 2). In this dataset, Greece possessed higher haplotypic (0.982) and remarkably higher nucleotide (0.030) diversity values compared to Central/West Europe. It is remarkable that Greece possesses slightly higher number of haplotypes than Central/West Europe (149 vs 130), despite the fact that its sample size is almost one third of Central/West Europe (287 vs 841 samples). In addition to this, according to the rarefaction curve the plateau has not been reached for the discovery rates of new haplotypes despite the additional extensive sampling in Greece, confirming the great diversity of Greek populations (S1 Fig). Furthermore, Greece possessed remarkably higher percentage of (population–specific) haplotypes per number of individuals compared to the rest of Europe.

**Fig 1:**
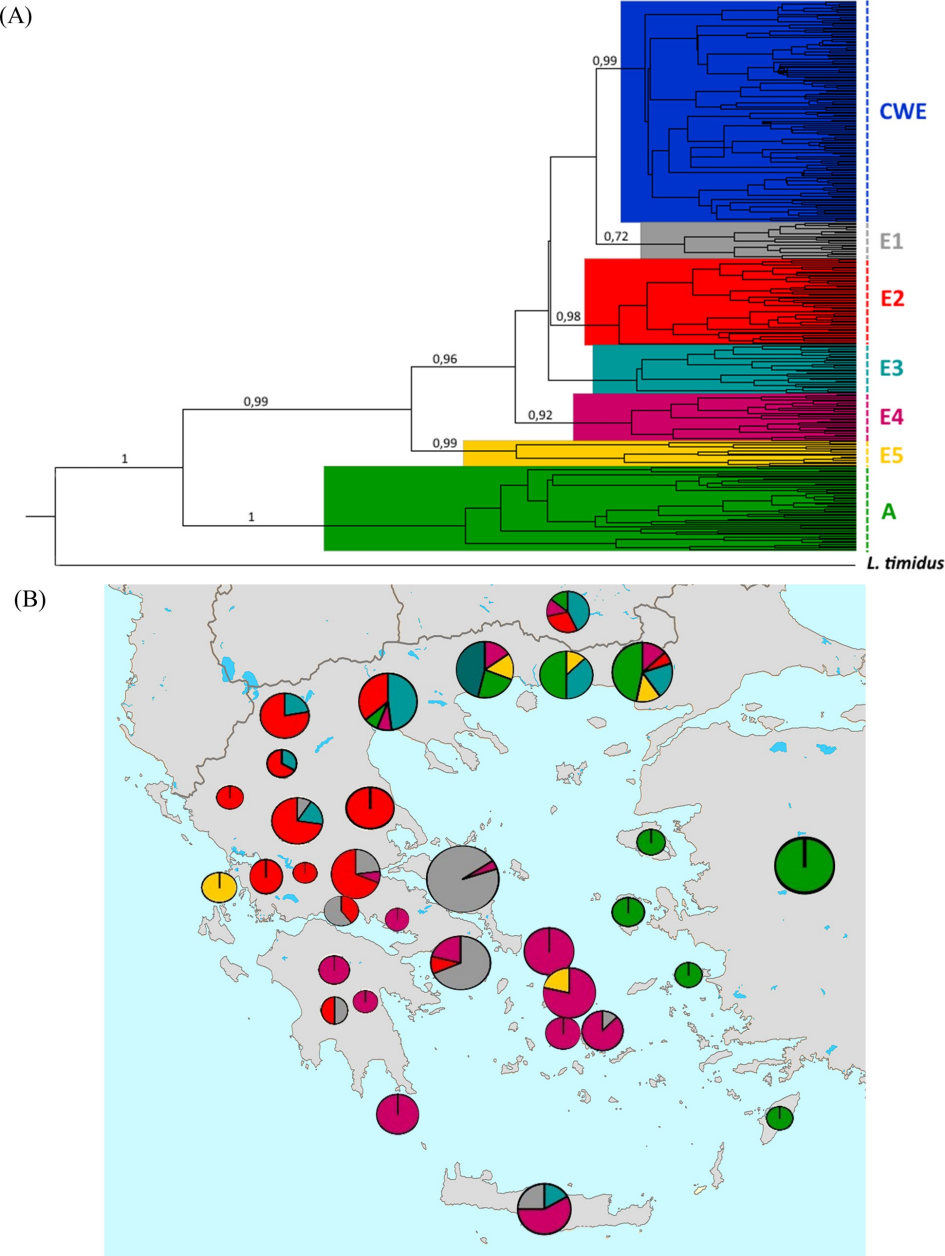
Phylogenetic tree (A) and distribution (B) of brown hare haplotypes in the Balkans. Subgroup A consists the “Anatolian” clade, while subgroups CWE and E1-E5 consist the “European” one. Each coloured circle in b represents the existence of individuals belonging to the coloured branches of the tree in a. The size of circles in b reveals the number of individuals studied from each area. Posterior probabilities >0.7 are shown (Map was modified from https://commons.wikimedia.org/wiki/File:Blanc_neutral_map_of_Greece.png, CC BY 2.5).

The Bayesian phylogenetic analysis (Fig 1) supported haplotype clustering in two distinct clades, a result consistent with previous studies [22, 24]. In the first clade, conventionally named “Anatolian” (A), haplotypes from Turkey, Cyprus, Israel, Bulgaria, Greece as well as an Italian haplotype were grouped. Only 4 haplotypes (two of them newly characterized) from the present study clustered in this clade (samples from Chios and Evros).

**Fig 2:**
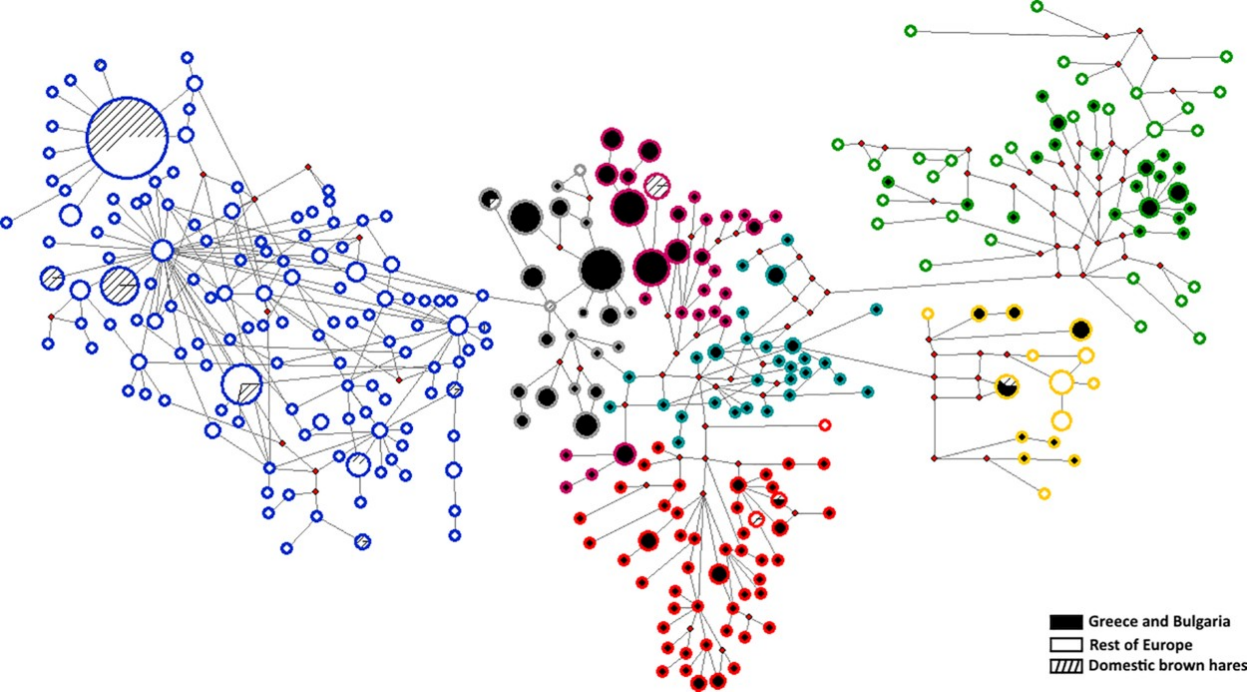
Median-Joining network of 335bp mtDNA brown hare haplotypes (326 haplotypes) in the full dataset. The proportional size of nodes indicates the frequency of haplotypes. The coloured line of circles corresponds to the coloured subgroups in the phylogenetic tree (Fig 1). Small red dots represent inferred haplotypes.

The second clade, the “European” one involved subgroups CWE and E1-E5 (Fig 1). This clustering is supported by high values of posterior probabilities (posterior probabilities >0.7 are shown, Fig 2). Subgroups E1-E5 were found mainly in Greece and Bulgaria. Most of the haplogroups presented specific geographic distribution within Greece. The E1 subgroup is mainly found in Evia island and Central Greece, E2 is spread over mainland Greece, the E3 mainly in North Greece, E4 mainly in Aegean islands (Cyclades and Crete), Central and South Greece. E5 is more widespread and observed in Northeastern Greece, Slovakia and Italy. The CWE subgroup includes the vast majority (122 out of 130) of haplotypes from countries of Central and Western Europe. Only 8 haplotypes from these areas were clustered in the E2 (one haplotype) and E5 (7 haplotypes) subgroups. No wild Greek haplotypes belong to subgroup CWE. The majority of reared individuals (97 out of 110 individuals, 13 out of 20 haplotypes) clustered in the CWE subgroup, while a low number of domestic haplotypes was included in groups E1, E2, E4 and E5 (7 haplotypes found only in 13 individuals).

In addition to the Bayesian analysis, a median-joining network was constructed to further elucidate the phylogenetic relationships among haplotypes (Fig 2). The grouping of haplotypes in the MJ network is in concordance with the clustering in Bayesian analysis. The network verified the existence of two distinct clades (Anatolian and European), which were separated by several mutational steps.

## Discussion

The combination of our data with previous studies has resulted in a broader and clearer depiction of brown hare genetic diversity distribution and sheds more light on the evolutionary factors that have shaped it.

### Importance of rear-edge Greek brown hare populations

Our data reveal that Greek populations present remarkably high genetic diversity. Although other studies have also illustrated Greece’s role as a biodiversity hotspot [2, 5, 17, 42, 43] it is the first time that such high diversity values are reported. The identification of numerous newly characterized haplotypes is important for revealing the evolutionary history of brown hare in Greece and Europe. These higher haplotype and nucleotide diversity values of Greek population as well as the high percentage of (population–specific) haplotypes per number of individuals, underline the existence of long-term favorable environmental conditions, under which these populations remained relatively stable and genetically variable [44]. The existence of a mosaic steppe in Greece [45, 46], has provided the appropriate environment for brown hares to survive and evolve.

The high diversity values in the Greek marginal populations combined with the low diversity of central European ones do not support the “centre-periphery hypothesis” which includes the idea that core populations present higher genetic diversity than the edge ones [47, 48, 49]. This pattern has also been observed in other species in Europe like the obligate myrmecophilus butterfly (*Maculinea arion*, [50]), which presented higher genetic diversity in one of the main European refugia, Italy. Such rear edge populations, manage to survive under suitable for species persistence conditions [51], maintaining a large fraction of their pre-glacial diversity and thus leading to the creation of independent refugia within the known refugia of southern European peninsulas [52]. This scenario has been demonstrated for a number of Greek species including wild boar [17], horned viper [43], alpine newt [42] and red deer [53] that have been reported to harbor genetically differentiated lineages from the rest of the Balkans.

The fact that high genetic diversity values of Greek populations were maintained for long time periods is also very well presented by the complex structure of the Bayesian phylogenetic analysis and the median-joining network, contradicting undeniably the “centre-periphery hypothesis”. The star-like phylogeny in CWE subgroup combined with the absence of geographic structure support the idea of rapid expansion and colonization of brown hare in Central and West Europe. On the contrary, the Greek haplotypes (mainly included in subgroups E1-E5) do not present very close phylogenetic relationships and there is a clear geographic patterning.

Although the concepts of geographic centrality, ecological marginality and centre of origin have been repeatedly presented in the literature, very few studies have focused on all these factors at the same time [44]. According to the centre-periphery hypothesis [54], species are more abundant in the centre of their geographic ranges, where climatic conditions are more optimal for their survival. As distance from the centre increases the environmental conditions become unsuitable. Subsequently, marginal populations may be more vulnerable to any changes and consequently more prone to extinction. Our results, however, support the view of Pironon et al. [44] who argue that it may, actually, be more accurate if we consider refugial populations (such as those from the southern Balkans, which are at a geographic rear-edge) as the central ones and populations from recently colonized areas (geographically central) as peripheral, and that therefore, the centre and the margin of populations cannot always be geographically delimited.

### Microgeographic structure of brown hares in Greece

The identification of haplotypes in newly sampled areas also gives us a more comprehensive view of the phylogeography of the species within Greece. As shown both by the Bayesian and network analyses different lineages (E1, E2, E3, E4) exist, with characteristic geographic structure. Similar results were obtained by Kasapidis et al. [22], who presented different clusters with specific geographic distribution. However, the present study reveals a much more extended geographic structure as we added a significant number of new samples and the clustering is strongly supported by high posterior probability values. These lineages may indicate the existence of different small refugia in Greece during the Last Glacial Maximum, supporting the “refugia within refugia” model. This model has been proven to fit many other species as well, for all three main refugia (Balkans, Iberia, Italy) [17, 43, 55].

Apart from historical interplay effects on the genetic structure of the species some other present day factors have to be considered in order to be more accurate in explaining current genetic data. Specifically, the present day isolation of different Greek populations cannot be only attributed to the geographical boundaries as there are no natural barriers between all sampling areas. According to [20], it can be explained by philopatric females that do not disperse in large distances. The maternal inheritance of mtDNA, used in the present study could overestimate the above differentiation. However, results of Antoniou et al. [31] are consistent in mitochondrial as well as nuclear markers, indicating that the philopatry of females does not affect the genetic structure of the species. Nevertheless, the study of Antoniou et al. [31] focused on a restricted area in Northeastern Greece and therefore a more thorough overview of demographic genetic variability in an extended region and the use of both mitochondrial and nuclear markers are essential, for safe conclusions.

Despite the high genetic variability of Greek populations, Aegean islands presented low haplotypic diversity values (Table 1), probably due to low population densities and thus reduced effective population sizes. It is noteworthy to mention that every different island studied (Andros, Paros, Tinos and Kythira) is represented by a unique haplotype, which is characteristic for this specific island, regardless of the number of samples analyzed. The long term geographic isolation of these populations, and related absence of gene flow could explain the presence of these unique haplotypes. One exception was noticed in the case of the island Naxos, in which two of the three haplotypes were shared with individuals from Evia. A contact of these areas in previous glacial periods is possible [56]. The low density of island populations and their low genetic variability render them more vulnerable to the introduction of foreign genomes in these areas, which can lead to deterioration of their genetic pool in short periods [57]. For this reason, it is important to protect them in the context of the protection of biodiversity of the species.

Considering the clustering of Chios with the Anatolian clade, it could be attributed to: i) human induced translocations of individuals from Turkey on this island and/or ii) vicariance when Chios separated from Turkey (about 9,000 years ago, [58]). However, any conclusion cannot be reached safely ​​without additional sampling and further analysis including archeological samples. This also applies for previously sampled populations from the islands of Lesvos and Samos, which also cluster with the Anatolian clade. Rhodes was not connected to the Asia Minor mainland since the end of the Pliocene or the beginning of the Pleistocene [59]. The presence of brown hare there is most probably due to human translocation.

The fact that many unique haplotypes have been found only in Greece, absent from Central Europe, supports the hypothesis that Greek populations were not involved in the northward expansion of the species. These data reinforce the concept that colonization of Europe took place starting from Central and Northern Balkan refugia [5], while the contribution of southern distinct populations was minimal. This scenario has been also proposed for species such as the grasshopper and the hedgehog and one possible explanation has been given from [5], indicating that northern Balkan populations of brown hare prevented the expansion of Greek ones from the southernmost distribution of the species.

Our data indicate that the north part of Greece was the region with the highest mtDNA diversity values (Table 1). This could be attributed to the coexistence of the two highly differentiated clades in the area of Thrace (European and Anatolian clade). According to previous studies [5], a well-known hybrid zone exists in North-Eastern Greece (Thrace) and South-Eastern Bulgaria/North-West Turkey [22, 24, 31] where the eastern (Anatolian) populations have managed to introgress. This also holds for other taxa (grasshopper, [56]) but it is not always consistent; for species including wild boar [17] and crested newt [60] it is clear that there is no such geographical overlap, whereas for the European ground squirrel, the overlap zone is limited [61]. It is clear that there is no common rule about the existence of an overlapping hybrid zone for all studied species. More research is needed in a variety of species in order to elucidate the mechanisms and the biological history of the species that can produce such differences, despite the shared habitat.

### Impact of restocking programs

During the last decades numerous restocking programs have taken place introducing foreign individuals of brown hare into several European countries [26, 28, 62]. In Greece, individuals have originated from breeding stations in Italy, Yugoslavia and Bulgaria [63]. These restocking programs were uncontrolled, in many cases, with unpredictable consequences on the historical distribution and genetic integrity of indigenous hare species [26–28]. The introduction of foreign genomes into local populations may influence its fitness [27].

The results of the present study indicate that the majority of reared individuals cluster with haplotypes from Central and Western Europe. Since release of such individuals in the environment is a common practice, this constitutes a potential risk to the domestic gene pool of the species. This is aggravated by the fact that farmed individuals can survive in the wild at least for a reproductive period, so it is possible that they transfer their genome [63]. However, it is also true that no Greek wild samples were found to belong to the CWE group, even though most reared individuals carry such haplotypes. This supports that no gene flow has yet been detected between reared and wild Greek populations. On the other hand, some haplotypes from domestic brown hares clustered with Greek wild haplotypes, implying that these individuals were collected from the wild and used as breeders. In any case, the use of endemic individuals as brood stock is a practice that should be followed in order to protect region specific diversity [28].

## Conclusion

Although during LGM, the Balkan peninsula acted as a major refugium for the colonization of the northern parts of Europe by brown hare [3, 5], the contribution of Greece seems to be minimal. Greek populations have retained a large fraction of their pre-glacial diversity and though restricted to a small refugial area, have managed to survive. Our analysis shows that a “refugia within refugia” model fits better brown hare evolution. These “peripheral” populations hold important abundant genetic variability relative to central populations and constitute significant units that deserve conservation priority [47]. For this reason, it is essential that any restocking program should be accompanied by genetic control of the individuals that will be released in the wild, in order to protect this genetic stock. This is indeed requested by the Greek state (Ministry Decision 98161/4136/29-9-2008 published in FEK 637/B/6-4-2009) for all stocked game animals.

## Supporting information

S1 Table Names of sampling localities for each region and corresponding geographic coordinates, number of samples and number of haplotypes. (DOCX)

S1 Fig Rarefaction curve for haplotype diversity of Greek brown hare individuals. (DOCX)

## References

[1] Clark PU, Dyke AS, Shakun JD, Carlson AE, Clark J, Wohlfarth B, et al The Last Glacial Maximum. Science. 2009;325: 710–714. 10.1126/science.1172873 19661421

[2] Hewitt GM. The genetic legacy of the Quaternary ice ages. Nature. 2000;405: 907–913. 10.1038/35016000 10879524

[3] Provan J, Bennett KD. Phylogeographic insights into cryptic glacial refugia. Trends Ecol Evol. 2008;23: 564–571. 10.1016/j.tree.2008.06.010 18722689

[4] Taberlet P, Fumagalli L, Wust-Saucy AG, Cosson JF. Comparative phylogeography and postglacial colonization routes in Europe. Mol Ecol. 1998;7: 453–464. 9628000 10.1046/j.1365-294x.1998.00289.x

[5] Hewitt GM. Post-glacial recolonization of Europe. Biol J Linn Soc. 1999;68: 87–112.

[6] Bilgin R. Back to the Suture: The Distribution of Intraspecific Genetic Diversity in and Around Anatolia. Int J Mol Sci. 2011;12(6): 4080–4103. 10.3390/ijms12064080 21747726 PMC3131610

[7] Randi E. Phylogeography of South European mammals In: Weiss S, Ferrand N, editors. Phylogeography in Southern European refugia: evolutionary perspectives on the origins and conservation of European biodiversity Dordrecht: Springer; 2007 p. 101–126,

[8] Niedziałkowska M. Phylogeography of European moose (*Alces alces*) based on contemporary mtDNA data and archaeological records. Mamm Biol. 2017;84: 35–43.

[9] Stewart JR, Lister AM. Cryptic northern refugia and the origins of the modern biota. Trends Ecol. Evol. 2001;16: 608–613.

[10] Parducci L, Jørgensen T, Tollefsrud M, Elverland E, Alm T, Fontana SL. et al Glacial Survival of Boreal Trees in Northern Scandinavia. Science. 2012;335: 1083–1086. 10.1126/science.1216043 22383845

[11] Schmitt T, Varga Z. Extra-Mediterranean refugia: The rule and not the exception? Front Zool. 2012;9(1): 22 10.1186/1742-9994-9-22 22953783 PMC3462695

[12] Kotlik P, Deffontaine V, Mascheretti S, Zima J, Michaux JR, Searle JB. A northern glacial refugium for bank voles (*Clethrionomys glareolus*). PNAS 2006;103: 14860–14864. 10.1073/pnas.0603237103 17001012 PMC1595441

[13] Sommer RS, Nadachowski A. Glacial refugia of mammals in Europe: evidence from fossil records. Mamm. Rev. 2006;36: 251–265.

[14] McDevitt AD, Zub K, Kawałko A, Oliver MK, Herman JS, Wójcik JM. Climate and refugial origin influence the mitochondrial lineage distribution of weasels (*Mustela nivalis*) in a phylogeographic suture zone. Biol. J. Linn. Soc. 2012;106: 57–69.

[15] Weiss S, Ferrand N. Phylogeography of southern European refugia Springer 2007.

[16] Centeno-Cuadros A, Delibes M, Godoy JA. Phylogeography of southern water vole (*Arvicola sapidus*): evidence for refugia within the Iberian glacial refugium. Mol Ecol. 2009;18: 3652–3667. 10.1111/j.1365-294X.2009.04297.x 19674304

[17] Alexandri P, Triantafyllidis A, Papakostas S, Chatzinikos E, Platis P, Papageorgiou N, et al The Balkans and the colonization of Europe: the post-glacial range expansion of the wild boar, *Sus scrofa*. J Biogeogr. 2012; 39: 713–723.

[18] Abellán P, Svenning JC. Refugia within refugia–patterns in endemism and genetic divergence are linked to Late Quaternary climate stability. Biol J Linn Soc. 2014;113: 13–28.

[19] Flux JEC, Angermann R. Rabbits, hares and pikas. Status survey and conservation action plan In Chapman JA, Flux JEC, editors. The hares and jackrabbits. Gland, Switzerland, and Cambridge, UK: IUCN/SSC Lagomorph Specialist Group; 1990 p 61–94.

[20] Hulbert IAR, Iason GR, Elston DA, Racey PA. Home-range sizes in a stratified upland landscape of two lagomorphs with different feeding strategies. J Appl Ecol. 1996;33: 1479–1488.

[21] Misiorowska M, Wasilewski M. Survival and causes of death among released brown hares (*Lepus europaeus* Pallas, 1778) in Central Poland. Acta Theoriol. 2012;57: 305–312. 10.1007/s13364-012-0081-1 PMC3443339 23002287

[22] Kasapidis P, Suchentrunk F, Magoulas A, Kotoulas G. The shaping of mitochondrial DNA phylogeographic patterns of the brown hare (*Lepus europaeus*) under the combined influence of Late Pleistocene climatic fluctuations and anthropogenic translocations. Mol Phylogenet Evol. 2005;34: 55–66. 10.1016/j.ympev.2004.09.007 15579381

[23] Fickel J, Hauffe HC, Pecchioli E, Soriguer R, Vapa L, Pitra C. Cladogenesis of the European brown hare (*Lepus europaeus* Pallas, 1778). Eur J Wildl Res. 2008;54: 495–510.

[24] Stamatis C, Suchentrunk F, Moutou KA, Giacometti M, Haerer G, Djan M, et al Phylogeography of the brown hare (*Lepus europaeus*) in Europe: a legacy of south-eastern Mediterranean refugia? J Biogeogr. 2009;36: 515–528.

[25] Dermitzakis DM, Papanikolaou DJ. Paleogeography and geodynamics of the Aegean region during the Neogene. Ann Geol Pays Hell. 1981;30: 245–289.

[26] Flux JEC. Intoduction to taxonomic problems in hares. Acta Zool Fenn. 1983;174: 7–10.

[27] Thulin CG, Jaarola M, Tegelstrom H. The occurrence of mountain hare mitochondrial DNA in wild brown hares. Mol Ecol. 1997;6: 463–467. 9161014 10.1046/j.1365-294x.1997.t01-1-00199.x

[28] Pierpaoli M, Riga F, Trocchi V, Randi E. Species distinction and evolutionary relationships of the Italian hare (*Lepus corsicanus*) as described by mitichondrial DNA sequencing, Mol Ecol. 1999;8: 1805–1817. 10620225 10.1046/j.1365-294x.1999.00766.x

[29] Hillis D, Moritz C, Mable BK. Molecular systematic Nucleic acids IV: sequencing and cloning. Sinauer Associates, Sunderland, MA 1996 p. 321–381.

[30] Thomson JD, Higgins DG, Gibson TJ. CLUSTAL W: improving the sensitivity of progressive multiple sequence alignment through sequence weighting, position specific gap penalties and weight matrix choice. Nucleic Acids Res. 1994;22: 4673–4680. 7984417 10.1093/nar/22.22.4673 PMC308517

[31] Antoniou A, Magoulas A, Platis P, Kotoulas G. Assessing the genetic landscape of a contact zone: the case of European hare in northeastern Greece. Genetica. 2013;141: 23–40. 10.1007/s10709-013-9703-z 23381134

[32] Guindon S, Gascuel O. A simple, fast and accurate algorithm to estimate large phylogenies by maximum likelihood. Syst. Biol. 2003;52: 696–704. 14530136 10.1080/10635150390235520

[33] Darriba D, Taboada GL, Doallo, Posada D. jmodeltest 2: more models, new heuristics and parallel computing. Nat methods, 2012; 9(8): 772–772. 10.1038/nmeth.2109 PMC4594756 22847109

[34] Tavaré S. Some probabilistic and statistical problems in the analysis of DNA sequences. In: Miura R M, editor. Lectures on mathematics in the life sciences. 1985;17: 57–86.

[35] Yang Z. Maximum likelihood phylogenetic estimation from DNA sequences with variable rates over sites: approximate methods. J. Mol. Evol. 1994;39: 306–314. 7932792 10.1007/BF00160154

[36] Drummond AJ, Suchard MA, Xie D, Rambaut A. Bayesian phylogenetics with BEAUti and the BEAST 1.7. Mol Biol Evol. 2012;29: 1969–1973. 10.1093/molbev/mss075 22367748 PMC3408070

[37] Rambaut A, Suchard MA, Xie D, Drummond AJ. Tracer v1.6. Available from http://beast.bio.ed.ac.uk/Tracer. 2014.

[38] Rambaut A, Drummond AJ. FigTree v1.3.1 Institute of Evolutionary Biology, University of Edinburgh: Edinburgh, UK (http://tree.bio.ed.ac.uk/software/figtree/). 2010.

[39] Bandelt HJ, Forster P, Ro¨hl A. Median-joining networks for inferring intraspecific phylogenies. Mol Biol Evol. 1999;16: 37–48. 10.1093/oxfordjournals.molbev.a026036 10331250

[40] Librado P, Rozas J. DnaSP v5: a software for comprehensive analysis of DNA polymorphism data. Bioinformatics. 2009;25: 1451–1452. 10.1093/bioinformatics/btp187 19346325

[41] Holland S. RarefactWin. Available from: http://strata.uga.edu/software/.

[42] Sotiropoulos K, Eleftherakos K, Dzukić G, Kalezić ML, Legakis A, Polymeni RM. Phylogeny and biogeography of the alpine newt *Mesotriton alpestris* (Salamandridae, Caudata), inferred from mtDNA sequences. Mol Phylogenet Evol. 2007;45: 211–226. 10.1016/j.ympev.2007.03.012 17467298

[43] Ursenbacher S, Schweigher S, Tomović L, Crnobrnja-Isailović J, Fumagalli L, Mayer W. Molecular phylogeography of the nose-horned viper (*Vipera ammodytes*, Linnaeus (1758)): evidence for high genetic diversity and multiple refugia in the Balkan peninsula. Mol Phylogenet Evol. 2008;46: 1116–1128. 10.1016/j.ympev.2007.11.002 18267369

[44] Pironon S, Papuga G, Villellas J, Angert AL, García MB, Thompson JD. Geographic variation in genetic and demographic performance: new insights from an old biogeographical paradigm. Biol Rev. 2017;92: 1877–1909. 10.1111/brv.12313 27891813

[45] Bennett KD, Tzedakis PC, Willis KJ. Quaternary refugia of north European trees. J. Biogeogr. 1991;18: 103–115.

[46] Geraga M, Tsaila-Monopolis S, Ioakim C, Papatheodorou G, Ferentinos G. Evaluation of palaeoenvironmental changes during the last 18,000 years in the Myrtoon basin, SW Aegean Sea. Palaeogeogr Palaeoc. 2000;156: 1–17.

[47] Hampe A, Petit RJ. Conserving biodiversity under climate change: the rear edge matters. Ecol Lett. 2005;8: 461–467. 10.1111/j.1461-0248.2005.00739.x 21352449

[48] Eckert CG, Samis KE, Lougheed SC. Genetic variation across species’ geographical ranges: the central–marginal hypothesis and beyond. Mol Ecol 2008;17: 1170–1188. 10.1111/j.1365-294X.2007.03659.x 18302683

[49] Munwes I, Geffen E, Roll U, Friedmann A, Daya A, Tikochinski Y, et al The change in genetic diversity down the core-edge gradient in the eastern spadefoot toad (*Pelobates syriacus*). Mol Ecol 2010;19: 2675–2689. 10.1111/j.1365-294X.2010.04712.x 20561190

[50] Patricelli D, Sielezniew M, Ponikwicka-Tyszko D, Ratkiewicz M, Bonelli S, Barbero F, et al Contrasting genetic structure of rear edge and continuous range populations of a parasitic butterfly infected by *Wolbachia*. *BMC Evol Biol*. 2013;13: 14 10.1186/1471-2148-13-14 23331872 PMC3558474

[51] Tzedakis PC, Lawson IT, Frogley MR, Hewitt GM, Preece RC. Buffered tree population changes in a Quaternary refugium: evolutionary implications. Science 2002;297: 2044–2047. 10.1126/science.1073083 12242441

[52] Gómez A, Lunt DH. Refugia within refugia: Patterns of phylogeographic concordance in the Iberian Peninsula In Weiss S, Ferrand N, editors. Phylogeography of southern European refugia. Dordrecht: Springer. 2007 p. 155–188

[53] Karaiskou N, Tsakogiannis A, Gkagkavouzis K, Operator of Parnitha National Park, Papika S, Latsoudis P, et al Greece: A Balkan Subrefuge for a remnant red deer (*Cervus elaphus*) population. J Hered. 2014;105(3): 334–344. 10.1093/jhered/esu007 24558101

[54] Sagarin RD, Gaines SD, Gaylord B. Moving beyond assumptions to understand abundance distributions across the ranges of species. Trends Ecol. Evol., 2006;21: 524–530. 10.1016/j.tree.2006.06.008 16815588

[55] Yannic G, Pellissier L, Dubey S, Vega R, Basset P, Mazzotti S, et al Multiple refugia and barriers explain the phylogeography of the Valais shrew, *Sorex antinorii*. Biol J Linn Soc. 2012;105: 864–880.

[56] Lykousis V. Sea level changes and shelfbreak prograding sequences during the last 400 ka in the Aegean margins: subsidence rates and palaeogeographic implications. Cont. Shelf Res. 2009;29: 2037–2044.

[57] Ricklefs RE, Lovette IJ. The roles of island area *per se* and habitat diversity in the species–area relationships of four Lesser Antillean faunal groups. J Anim Ecol. 1999;68: 1142–1160.

[58] Andel TM, Shackleton JC. Late Paleolithic and Mesolithic coastlines of Greece and the Aegean. J. Field Archaeol. 1982;9: 445–454.

[59] Dermitzakis DM. Paleogeography, geodynamic processes and event stratigraphy during the late Cenozoic oft he Aegean area. Accad Naz Lincei. 1990;85: 263–288.

[60] Wielstra B, Themudoa GE, Güçlüc Ö, Olgunc K, Poyarkova NA, Arntzena JW. Cryptic crested newt diversity at the Eurasian transition: The mitochondrial DNA phylogeography of Near Eastern *Triturus newts*. Mol Phylogenet Evol. 2010;56: 888–896.61. 10.1016/j.ympev.2010.04.030 20435147

[61] Kryštufek B, Bryja J, Bužan EV. Mitochondrial phylogeography of the European ground squirrel, *Spermophilus citellus*, yields evidence on refugia for steppic taxa in the southern Balkans. Heredity. 2009;103: 129–135. 10.1038/hdy.2009.41 19384339

[62] Perez-Suarez G, Palacios F, Boursot P. Speciation and paraphyly in western mediterranean hares (*Lepus castroviejoi*, *L*. *europaeus*, *L*. *granatensis*, and *L*. *capensis*) revealed by mitochondrial DNA phylogeny. Biochem Genet. 1994;32: 423–436. 7748159 10.1007/BF00566063

[63] Mamuris Z, Sfougaris AI, Stamatis C. Genetic structure of Greek brown hare (*Lepus europaeus*) populations as revealed by mtDNA RFLP-PCR analysis: implications for conserving genetic diversity. Biol Conserv. 2001;101: 187–196.

