# Supplementary material for: Genetic analyses of brown hare (Lepus europaeus) support limited migration and translocation of Greek populations
Source: PLoS One. 2018 Oct 31;13(10):e0206327. doi: 10.1371/journal.pone.0206327 (PMC6209229; doi:10.1371/journal.pone.0206327)
Supplement: S1 Table — (DOCX) [file pone.0206327.s001.docx]

| Region | Locality | Geographic Coordinates | | Number of samples | Number of haplotypes |
| --- | --- | --- | --- | --- | --- |
|  |  | Latitude | Longitude |  |  |
| North Greece | Evros | 41.2225681 | 26.3012695 | 12 | 7 |
|  | Grevena | 40.0856892 | 21.4284718 | 4 | 4 |
| Central – South Greece | Arkadia | 37.5315099 | 22.1978759 | 1 | 1 |
|  | Attiki | 38.0113122 | 23.8265991 | 19 | 11 |
|  | Evia | 38.5546093 | 23.8211059 | 47 | 12 |
|  | Evritania | 38.9743572 | 21.6403198 | 1 | 1 |
|  | Fokida | 38.4879946 | 22.2610473 | 5 | 3 |
|  | Fthiotida | 38.8782049 | 22.3489379 | 13 | 10 |
|  | Trikala | 39.5506476 | 21.7254638 | 2 | 2 |
|  | Viotia | 38.3545803 | 23.0850219 | 1 | 1 |
| Central Aegean Islands | Andros | 37.8409114 | 24.8623237 | 13 | 2 |
|  | Kithira | 36.2531331 | 22.9833984 | 5 | 2 |
|  | Naxos | 37.0600104 | 25.4707567 | 7 | 3 |
|  | Paros | 37.0651048 | 25.1921674 | 6 | 2 |
|  | Tinos | 37.6014025 | 25.1541436 | 14 | 2 |
| Eastern Aegean Islands | Chios | 38.3997966 | 26.0403442 | 1 | 1 |
| Ionian Islands | Lefkada | 38.7064706 | 20.6417326 | 3 | 1 |
