# Supplementary material for: Genetic analyses of brown hare (Lepus europaeus) support limited migration and translocation of Greek populations
Source: PLoS One. 2018 Oct 31;13(10):e0206327. doi: 10.1371/journal.pone.0206327 (PMC6209229; doi:10.1371/journal.pone.0206327)
Supplement: Supplementary file 2 [file pone.0206327.s002.docx]

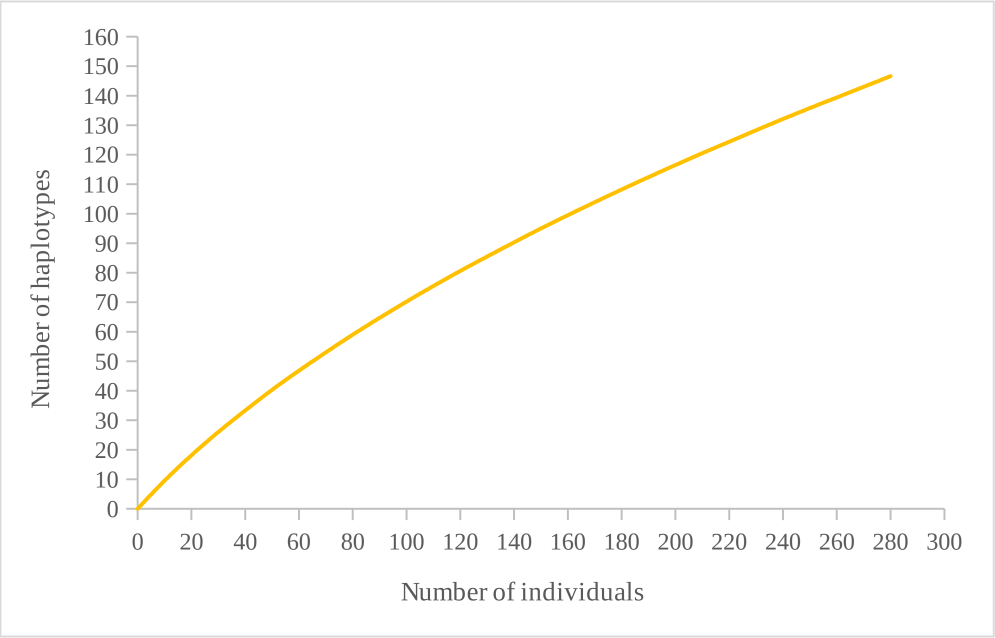
Number of haplotypes
160
150
140
130
120
110
100
90
80
70
60
50
40
30
20
10
0
0
20
40
60
80
100
120
140
160
180
200
220
240
260
280
300
Number of individuals
